# The genetic landscape of substance use disorders

**DOI:** 10.1038/s41380-024-02547-z

**Published:** 2024-05-29

**Authors:** Zachary F. Gerring, Jackson G. Thorp, Jorien L. Treur, Karin J. H. Verweij, Eske M. Derks

**Affiliations:** 1 Translational Neurogenomics Laboratory, Mental Health and Neuroscience, QIMR Berghofer Medical Research Institute, Brisbane, QLD Australia; 2 Department of Psychiatry, Amsterdam UMC, location University of Amsterdam, Amsterdam, the Netherlands

**Keywords:** Genetics, Addiction

## Abstract

Substance use disorders represent a significant public health concern with considerable socioeconomic implications worldwide. Twin and family-based studies have long established a heritable component underlying these disorders. In recent years, genome-wide association studies of large, broadly phenotyped samples have identified regions of the genome that harbour genetic risk variants associated with substance use disorders. These regions have enabled the discovery of putative causal genes and improved our understanding of genetic relationships among substance use disorders and other traits. Furthermore, the integration of these data with clinical information has yielded promising insights into how individuals respond to medications, allowing for the development of personalized treatment approaches based on an individual’s genetic profile. This review article provides an overview of recent advances in the genetics of substance use disorders and demonstrates how genetic data may be used to reduce the burden of disease and improve public health outcomes.

## Introduction

Substance use disorders (SUDs) encompass a range of common and heritable psychiatric disorders that result from a complex interplay of genetic and environmental factors. Together, SUDs affect millions of people and account for a significant proportion of the global burden of disease. For example, alcohol use disorder (AUD) affected over 100 million people in 2019 and was responsible for around 160,000 deaths, while opioid use disorder (OUD) affected around 21 million people and contributed to more than 88,000 deaths [1]. Substance use disorders also increase the risk of other leading contributors of morbidity and mortality, such as poisoning, suicide and self-inflicted injuries related to alcohol and opioid use, as well as chronic diseases like chronic obstructive pulmonary disease related to tobacco use [2]. Understanding the genetic causes of SUDs will facilitate the development of more effective treatments and prevention strategies, helping to alleviate the global burden of SUDs.

Genome-wide association studies (GWAS) have emerged as a powerful tool to identify genetic variants associated with SUDs. This approach has provided valuable insights into the genetic architecture of SUDs, revealing genomic regions and candidate causal genes that contribute to susceptibility. This review will discuss recent advancements in the genetics of SUDs, including (i) the identification of robust (replicable) risk variants; (ii) how these risk variants have been used to identify novel genes and disease mechanisms; (iii) the use of GWAS for polygenic scores (PGS); (iv) how risk variants have been used to establish causal associations both within SUDs and between SUDs and other disorders; and (v) and how these genetic factors influence an individual’s response to drugs. We will conclude the review with discussion on clinical and therapeutic implications of genetic findings for SUDs.

### Genetic variation underlying major substance use disorders

**Fig. 1:**
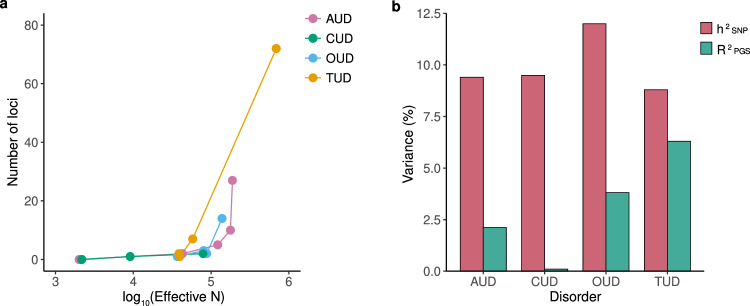
A summary of GWAS findings for major substance use disorders. **a** Number of genomic loci associated with substance use disorders verse GWAS sample size (see Supplementary Table 1). **b** SNP-based heritability (h^2^snp) and variance explained by polygenic scores (*R*^2^ PGS) for substance use disorders (extracted from refs. [9, 18, 26, 42, 43]). Notes: AUD alcohol use disorder, CUD cannabis use disorder, OUD opioid use disorder, TUD tobacco use disorder, Effective N effective sample size.

Genome-wide association studies have been central to the identification of common (minor allele frequency [MAF] > 0.01) SNPs associated with SUDs. In addition, it is recognized that rare genetic variants, including rare SNPs (MAF < 0.01), copy number variants (CNVs), and structural variants (SVs), may also play a significant role in the susceptibility to SUDs. Because statistical power increases with higher MAF, common SNPs underlying SUDs are routinely identified through GWAS meta-analyses of increasingly large, broadly phenotyped biobanks, such as the Million Veteran Program (MVP) [3] and the UK Biobank (UKB) [4]. Rare variants, defined by their low frequency in the population, are more challenging to detect and analyze. However, they are known to exert a larger effect compared to common variants and have the potential to advance our understanding of risk genes and biological pathways underlying SUDs [5]. This section summarizes known genetic risk factors underlying SUDs, with particular emphasis on recently published results. We provide a summary of recent genetic findings for SUDs in Fig. 1, including the number of identified loci (1a) and the amount of variation explained by common variants (SNP-based heritability, or h^2^snp) and polygenic scores (1b).

### Alcohol use disorder

The heritability of AUD from twin and family-based studies is around 50% [6], and the estimated h^2^snp is between 5.6% to 10.0% [7]. Early GWAS of Alcohol Dependence and measures of problematic drinking collectively identified more than 10 risk variants that mapped to several risk genes, most notably the alcohol dehydrogenase genes *ADH1B* (Alcohol Dehydrogenase 1B [class I], Beta Polypeptide) and *ADH1C* (Alcohol Dehydrogenase 1 C [class I], Gamma Polypeptide) [8]. The largest available meta-analysis of problematic alcohol use (PAU), which combined both AUD and problematic drinking data from the Million Veteran Program (MVP), the UK Biobank, and the Psychiatric Genomics Consortium (PGC), identified 29 independent risk variants, 19 of which were novel [9]. These risk variants mapped to 66 genes, including previously implicated *DRD2* (Dopamine Receptor D2), along with additional genes encoding alcohol dehydrogenase enzymes *ADH4*, *ADH5*, and *ADH7*. Functional analyses of these genes found significant enrichment of genetic signal for problematic alcohol use in several brain tissues and neural cell types. A recent multivariate GWAS jointly modelled the genetic effects of four SUDs (AUD, CUD, OUD, and TUD) to identify both shared and unique (i.e., substance-specific) genetic effects underlying each disorder [10]. The study identified 9 independent SNPs specifically associated with problematic drinking; as expected, the most significant SNP effect mapped to the gene *ADH1B*, and biological pathway enrichment analysis implicated alcohol metabolism pathways.

There have been recent efforts to identify sources of structural variants underlying alcohol use disorder, however statistical power is limited with current sample sizes. A meta-analysis of five cohorts investigating AUD identified nine regions of CNVs, including a suggestive association on chromosome 5q21.3 [11]. Further replication studies confirmed the presence of these CNV regions, and additional analyses focusing on pathways and gene-drug interactions revealed the involvement of the mitogen-activated protein kinase signalling pathway and specific drugs related to AD biology or treatment. In a separate study conducted on Mission Indian families, a whole genome scan also uncovered genetic linkage between chromosome 5q21.3 and “craving for alcohol.” [12] These findings suggest genomic structural variation may play a role in the risk for alcohol dependence, although larger studies will be required to characterise the global CNV burden between cases and controls.

### Cannabis use disorder

Although cannabis use is prevalent, most users do not progress to cannabis use disorders. Cannabis use disorder (CUD) has a moderate heritability of ~0.5–0.6, which slightly exceeds the estimates for cannabis use and initiation phenotypes (*h*^2^ = ~0.4–0.5) [13]. Early GWASs of cannabis use disorders identified genome-wide significant variants, however there was poor replication between studies due in part to small sample sizes (the largest study included 51,372 individuals with 2387 cases) and heterogeneity among samples [14–17]. Only one locus, associated with a cis-eQTL for *CHRNA2* (Cholinergic Receptor Nicotinic Alpha 2 Subunit), was consistently identified as robustly associated with CUD.

A GWAS meta-analysis of 20,916 CUD cases and 363,116 controls replicated the *CHRNA2* locus and identified a novel association in *FOXP2* (Forkhead box protein P2) [18]. A cross-ancestry multivariate GWAS of substance use disorders suggested the signal for the*CHRNA2* is CUD-specific (i.e., the locus is not shared with other SUDs) [10]. On the other hand, the same multivariate analysis found the *FOXP2* locus was associated with both CUD and problematic tobacco use (PTU) in Europeans and CUD and OUD in African Americans, suggesting pleiotropic (i.e., not CUD-specific) SNP effects drive this association.

A recent study used whole genome sequencing (WGS) to investigate the relationship between low-frequency (MAF < 0.02) variants and DSM-IV defined CUD in a community-based sample of Native Americans and a European ancestry family-based sample [19]. The analysis used a set-based approach, where separate analyses were performed for low-frequency variants in coding regions followed by regulatory elements. A single genome-wide significant association was found in the coding region of *C1orf110* and the regulatory region in the *MEF2B* gene in a meta-analysis of both samples. While these data point to the contribution of rare variants to the heritability of CUD, further studies in large, population-based cohorts with replication in independent samples is required.

### Tobacco use disorder

Genetic factors play a significant role throughout the stages of cigarette smoking and tobacco (nicotine) use disorder (TUD). Studies suggest that there is a considerable range of heritability estimates for nicotine dependence, typically falling between approximately 0.30 and 0.70 [20, 21]. The wide range of heritability estimates may be influenced by the choice of TUD assessment, where the Fagerström Tolerance Questionnaire (FTQ) and the Fagerström Test for Nicotine Dependence (FTND) may produce different results to the gold standard DSM-IV and DSM-5 (Diagnostic and Statistical Manual of Mental Disorders) [22, 23].

A GWAS meta-analysis involving 38,602 smokers across 15 studies using the FTND and information on cigarettes per day, identified 6 replicable associations, including previously known associations with the CHRNA5-CHRNA3-CHRNB4 genes. A novel intronic variant in DNMT3B (rs910083) was linked to an increased risk of nicotine dependence, particularly severe dependence, and was replicated in the UK Biobank using a severe dependence phenotype. The integration of tissue-specific methylation and expression data found rs910083/DNMT3B was also a cis-methylation quantitative trait locus (QTL) and a cis-expression QTL, suggesting its role in influencing DNMT3B methylation levels in foetal brain and gene expression in adult cerebellum [24].

A follow-up study in 58,000 smokers of European and African ancestry using the FTND questionnaire identified five genome-wide significant loci, including previously undiscovered variants near MAGI2/GNAI1 and TENM2 [25]. Both variants influence the expression of nearby genes. The integration of tissue-specific gene expression data found rs2714700/MAGI2-AS3 affects gene expression in the hippocampus, while rs1862416/TENM2 affects expression in the lung. Interestingly, the variant rs2714700 consistently showed association with the heaviness of smoking index from the UK Biobank, while rs1862416 did not. This suggests secondary analyses based on the quantity of tobacco consumed may provide additional insight into the genetic effects on tobacco use dependence.

The availability of electronic health record (EHR) data with linked SNP genotype data has the potential to greatly increase samples sizes without the need to prospectively recruit individuals with clinical diagnoses. A recent multi-ancestry GWAS meta-analysis of TUD combined data from 898,680 individuals of European, African American, and Latin American ancestry from 5 biobanks under the PsycheMerge partnership with a further 244,890 individuals from the UK Biobank [26]. The study identified 97 genome-wide significant lead SNPs located in 72 independent loci, all of which were previously reported in much larger GWAS of smoking-related phenotypes (e.g., GWAS and Sequencing Consortium of Alcohol and Nicotine use [GSCAN] smoking initiation and cessation) [27]. This supports the use of EHRs in identifying replicable associations with smaller sample sizes. The analysis also provided support for nicotinic acetylcholine receptor genes as risk genes for smoking-related traits as well as being involved in dopaminergic transmission, including *DRD2*, *DBH*, *KDM4A*, *PDE4B*, and *NCAM1*.

Several studies have investigated low frequency and rare exonic variants in tobacco use, but have largely failed to identify replicable, exome-wide significant results outside of nicotinic cholinergic receptor genes [28–31]. A recent exome-wide association study of rare (MAF < 0.01) variants found a protective association with *CHRNB2* (Neuronal acetylcholine receptor subunit beta-2), where carriers of rare predicted loss of function or deleterious missense variants have a 35% lower odds of heavy smoking [32] (defined as at least 10 cigarettes per day either currently or formerly) and 18% lower odds of ever smoking. A leave-one-variant-out analysis identified a deleterious missense variant in *CHRNB2*, which was later validated in an independent cohort. Importantly, the authors found nominally significant enrichment of protective associations of the variant with other phenotypes, including substance use disorders (excluding alcohol), suggesting a genetic effect on both tobacco consumption and dependency phenotypes.

### Opioid use disorder

Approximately 50% of the variability in opioid dependence is due to additive genetic effects [33], with around 38% of the variability accounted for by genetic risk factors unique to opioid use [34]. Several GWAS with modest sample sizes (the largest comprising 10,544 cases) have reported genome-wide significant loci [35–39], however none were replicated using an independent sample. A GWAS of 82,707 European American individuals identified a coding variant in the *OPRM1* (opioid receptor mu 1) gene, which was later replicated in 2 independent cohorts [40] and further strengthened in a larger multi-trait GWAS of opioid addiction [41]. A subsequent meta-analysis of seven cohorts identified three genome-wide significant lead SNPs in a European ancestry meta-analysis, including variants in the *FURIN* and *OPRM1* genes [42]. Furthermore, a multi-trait analysis of GWAS (MTAG) combining OUD with AUD and CUD revealed 18 independent genome-wide significant loci, suggesting common (i.e., shared) genetic factors contribute to the development of multiple substance use disorders [42].

A recent cross-ancestry meta-analysis of 425,944 individuals in the MVP cohort used both stringent definitions of OUD (described by Zhou et al. [40]) and less stringent definitions which only required a single diagnostic code from the International Classification of Diseases (ICD-9 or ICD-10) for opioid abuse or dependence and identified 14 genome-wide significant loci [43]. In this study, a cross-ancestry meta-analysis of the less stringent OUD diagnosis in the MVP sample revealed 12 genome-wide significant variants, 3 of which replicated in a cross-ancestry GWAS meta-analysis of strictly defined OUD. These included variants in *OPRM1*, replicating the original MVP GWAS [40], in addition to variants in *FURIN* and near the gene *TSNARE1*. The authors found significant heritability enrichment of gene expression for OUD in multiple brain tissues previously associated with addiction. In addition, a transcriptome-wide association study found genes with differential gene expression underlying OUD in both brain and multiple peripheral tissues, such as adipose, gastrointestinal, and liver. This suggests OUD-related genetic variation may affect biological processes in both the brain and periphery.

The definition of cases (e.g., the number and type of diagnostic codes) and controls (e.g., the use of opioid-exposed, unexposed, and/or population-based controls), varies across OUD GWAS, which may decrease the generalizability of results. However, there are high genetic correlations across cohorts with different definitions of OUD cases and controls (rg > 0.9) [41], suggesting shared genetic effects contribute to OUD across a range of case/control definitions.

Little progress has been made on studies of rare variation underlying OUD, primarily due to the large sample sizes typically required to identify rare variants. The largest study to date used genotyping and copy number variation (CNV) calling methods in a sample of European–American and African American OUD cases and controls. Genome-wide association analysis of CNVs with OUD identified two deletions and one duplication that were significantly associated with OUD, including a chromosome 18q12.3 deletion with a protective effect [44].

### Genetic overlap between SUDs and with other complex traits

**Fig. 2:**
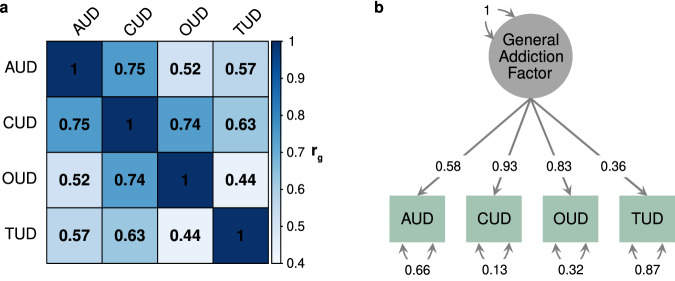
Pleiotropy between substance use disorders. **a** Genetic correlations between substance use disorders (extracted from refs. [25, 26, 138], which estimated correlations using linkage disequilibrium score regression). **b** Substance use disorders cluster to form a general addiction factor (path coefficients extracted from model reported in Hatoum et al. [10]., estimated using genomic structural equation modelling).

Consistent with high rates of comorbidity, there is substantial overlap in genetic risk factors between different substance use disorders. Recent studies have observed substantial pleiotropy at genome-wide [45], regional [46], and transcriptomic [47] levels. Genetic correlations range from ~0.45 (TUD and OUD) to ~0.70 (AUD and OUD) (see Fig. 2a) suggesting that while there is extensive overlap, there are also substance-specific genetic effects.

A recent study leveraged these correlations in a multivariate GWAS of four SUDs (AUD, TUD, CUD, and OUD), comprising over 1 million individuals. Using genomic structural equation modelling, the authors identified a general addiction risk factor (see Fig. 2b). A GWAS of the addiction factor identified 19 significant SNPs, the most significant of which was near *DRD2*, and functional enrichment analyses implicated pathways related to neural cells and immune cell processes. A separate study attempted to disentangle genetic effects underlying SUDs using exome-focussed genotyping of four SUDs (AUD, *n* = 4487; TUD, *n* = 4394; CUD, *n* = 954 and nonmedical prescription OUD, *n* = 346) in a large population-based sample (*n* = 36,309) [48]. An exome-wide association of common (MAF > 0.01) SNPs identified 53 experiment-wide significant SNPs associated with at least one SUD or a combined analysis of all four SUDs. A gene-based analysis of rare variants (MAF < 0.05) implicated 24 genes (using a nominal threshold of *P* < 10^-4^), all of which were previously implicated in large SUD GWAS with at least nominal (*P* < 0.05) significance. Finally, a “shared inheritance” (i.e., pleiotropic) analysis of all SUDs by gene-based association of rare alleles identified 9 genes associated with at least 2 SUDs. Collectively, these data suggest presence of widespread pleiotropy across SUDs.

There is also substantial pleiotropy between substance use disorders and psychiatric disorders (see Fig. 2), with the strongest overlap observed with ADHD, MDD, Anxiety, and schizophrenia. AUD has the most overlap with psychiatric disorders (mean |*r*_g_| = 0.32) while OUD has the least (mean |*r*_g_| = 0.18). While psychiatric disorders are generally positively genetically correlated with SUDs, OCD is noteworthy given its significant negative correlation with AUD (*r*_g_ = −0.35, se = 0.13) and TUD (*r*_g_ = −0.24, se = 0.05).

### Substance use phenotypes

As ascertaining large numbers of individuals with a diagnosed SUD is challenging, many studies have focused on broader, use-based phenotypes such as initiation, frequency, or quantity of use, which are easily assessed in large-scale cohorts and biobanks. These efforts are largely driven by consortia such as GSCAN [27, 49] and the International Cannabis Consortium (ICC) [50–52].

A recent large-scale, multi-ancestry GWAS (up to ~3.3 million individuals) by GSCAN included four tobacco use traits (smoking initiation, age of smoking regularly, smoking cessation, and cigarettes per day) and the alcohol use trait ‘drinks per week’ [27]. This study identified a remarkably large number of associated risk loci, including 1346 loci for smoking initiation and 496 for drinks per week. The largest cannabis-related genetic study to date by the ICC is a GWAS of lifetime cannabis use [52]. This identified 8 genome-wide significant SNPs and 35 genes, the strongest association with *CADM2*. A GWAS of ‘age at first cannabis use’ identified a single locus (*ATP2C2*) [50].

**Fig. 3:**
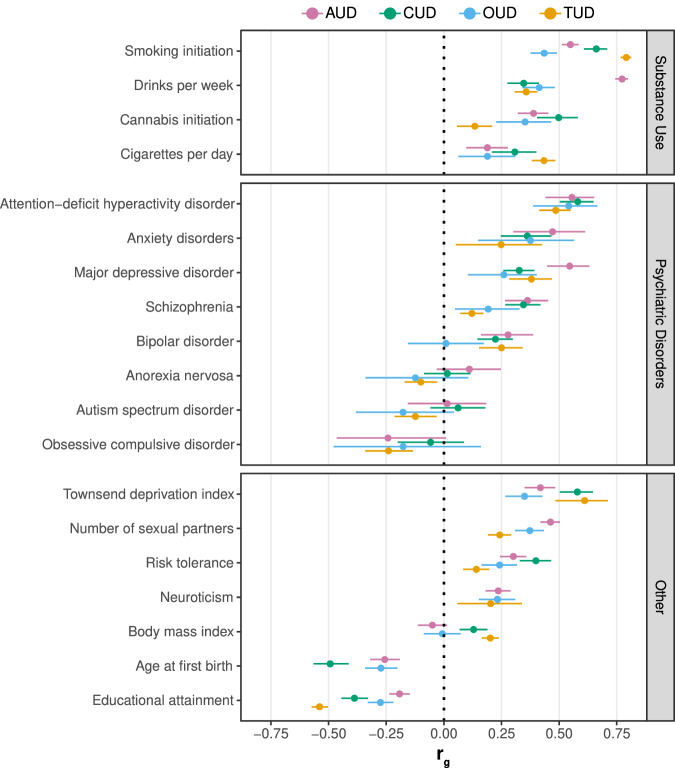
Genetic overlap between substance use disorders and other complex traits. Genetic correlations between alcohol use disorder (AUD), cannabis use disorder (CUD), opioid use disorder (OUD), tobacco use disorder (TUD) and a range of complex traits. Genetic correlations extracted from recent studies that applied linkage disequilibrium score regression (refs. [9, 18, 25, 26, 43, 138]). Error bars represent 95% confidence intervals.

These substance use phenotypes overlap moderately with dependence / disorder phenotypes (tobacco use and TUD *r*_g_ = ~ 0.4 − 0.8; alcohol quantity and AUD *r*_g_ = ~ 0.75; cannabis use and CUD *r*_g_ = ~ 0.50; see Fig. 3). This suggests there is considerable shared genetic etiology between use and dependence, and GWAS of use phenotypes can provide important biological insights into substance use disorders. But importantly, the imperfect overlap reinforces that substance use and dependence are different and GWAS of strictly defined SUDs are needed to dissect the distinct aspects that leads to substance dependence.

Overall, substance use phenotypes (such as initiation, frequency, and quantity) tend to have less genetic overlap with psychiatric disorders and related traits than dependence phenotypes. For example, analysis of different dimensions of alcohol use (alcohol consumption versus problematic consequences of drinking) in the Alcohol Use Disorders Identification Test (AUDIT) revealed a divergent pattern of genetic correlations with other psychiatric traits: problematic drinking was positively correlated with ADHD and MDD, while alcohol consumption was negatively correlated with these same disorders [53]. A similar pattern is also observed with tobacco, where use-based phenotypes [49] have significantly lower genetic correlations with psychiatric disorders than TUD [26].

Interesting, divergent patterns of association are also observed within different aspects of consumption (e.g., quantity vs. frequency). The genetic correlation between alcohol quantity and frequency in the UK Biobank was just 0.52 [54], and these measures display opposite patterns of association with indices of socioeconomic status, other substance use phenotypes, and psychiatric disorders. For example, alcohol frequency is negatively correlated with MDD, ADHD, and smoking initiation, while quantity is positively correlated with these same disorders. Taken together, these results suggest the presence of unique genetic factors underlying different types of consumption within the same disorder.

### Polygenic prediction of substance use disorders

Genetic effect estimates from genome-wide association studies can be used to calculate PGS that provide an indication of an individual’s genetic liability to a certain trait or disease. PGS can be calculated by multiplying the number of risk alleles a person carries with the SNP effect sizes from a GWAS of that phenotype and aggregating across all SNPs (taking LD into account). In research, these scores can then be used to validate the predictive power of the GWAS results, estimate associations with other traits, or test gene-environment interplay. Moreover, PGSs also hold potential for future clinical use.

In general, PGS only explain a small proportion of a trait’s variance, but they are valuable as they provide a measure of individual level genetic risk that can be used in analytical models. The validity to predict complex behavioural and psychiatric phenotypes has been well demonstrated for many traits [55]. For substance use disorders, current PGS explain approximately 2.1% for AUD [9], 3.8% for OUD [42], and 6.3% for TUD [26] (Fig. 1B). These estimates are lower than for some other psychiatric disorders, mostly due to the larger GWAS sample sizes for these other disorders. The predictive value of polygenic scores will grow when genome-wide effect estimates become more accurate once larger, more powerful GWASs become available. An important note is that PGS are most predictive in samples of similar ancestry as the source GWAS effect estimates are based on, due to differences in allele frequencies and LD between populations. The large-scale GWASs of SUDs are predominantly based on European ancestry participants, so PGS will be most powerful in European ancestry target samples.

Since the publications of the large-scale GWASs on AUD, CUD, and other substance use disorders, their summary statistics have been used to create PGS in independent samples to predict substance use, mental health, or other phenotypes. PGS for AUD were most often utilized in PGS analyses, while only very few studies included CUD or other substance use disorder PGS.

For AUD, most studies have found that AUD PGS (or PGS based on problematic alcohol use) significantly predict AUD and related alcohol use phenotypes [56–62], while a few other PGS analyses have not yielded significant results (e.g [63]. and [64] [in females only]). AUD PGS predicts an earlier age of onset for first substance use, regular use, the initiation of alcohol related problems, and alcohol dependence diagnosis [65]. AUD PGS have also been found to be positively associated with use of other substances [9, 60], mental health problems, including depression, anxiety disorder, bipolar disorder, ADHD, pathological gambling [9, 66, 67], myocardial infarction type 2 [68], nutrition intake [62], and neural connectivity in males [69] and negatively associated with cognition [60, 69]. Other studies showed no significant associations of AUD PGS with for instance epigenetic aging [70], timing of first marriage or likelihood to divorce [71], impulsivity [72], resilience [73] or AUD treatment outcome [74]. Some studies also investigated PGS associations in non-European ancestry, associations separately by sex, or tested for gene-environment interaction effects. For example, an AUD PGS from African ancestry individuals predicts age of regular use, alcohol dependence, and progression from regular use to alcohol dependence diagnosis [65]. However, most of these analyses yielded non-significant associations (e.g. [63, 64, 67, 75, 76]), likely due to the lack of statistical power.

With respect to CUD, there have only been a few PGS studies. Segura et al. [77] found that CUD PGS were significantly associated with cannabis use and monthly cannabis use at baseline, but not with age at initiation of cannabis use or with various measures related to the clinical course after a first-episode psychosis. In contrast, Cheng et al. [78] showed that PGS for CUD predicted bipolar disorder with psychotic experience but not bipolar disorder without psychotic experience.

Paul et al. [79] investigated the association between polygenic risk for substance use and cognition, and found no significant association between a CUD PGS with any of the measured cognition variables, whereas the PGS for lifetime cannabis use was positively associated with general ability, executive function, and learning/memory.

PGS for other substance use disorders showed mixed results. Two studies found that OUD PGS significantly predicted opioid use phenotypes ([60, 67] [only in the European, not the African ancestry, subsample]). Significant positive associations were also found for OUD PGS with other substance use related traits, negative associations with educational attainment and SES-related traits, as well as positive associations with several mental health traits (e.g. phenotypes related to conduct disorder and depression [60]), whereas in another study no significant associations were found between OUD PGS and a range of health-related phenotypes [67].

Vilar-Ribó, et al. [66]. tested whether the genetic liability to five SUD-related phenotypes was associated with ADHD and found that PGS for cocaine dependence and *ever addicted to illicit* drug did not yield significant associations with ADHD, while PGS for lifetime cannabis use, alcohol dependence and smoking initiation were significantly associated. Moreover, Hatoum et al. [10]. created a latent general addiction risk factor and found that the PGS based on this factor were associated with substance use disorders, psychopathologies, somatic conditions, and environments associated with the onset of addictions. In another study, a PGS based on substance misuse was significantly predictive of COVID-19 [80].

### Causes and consequences of substance use disorders

Identifying modifiable risk factors for disease is of particular interest in medicine and epidemiology, as it can inform preventive efforts and improve treatment. For many important health questions, it is not feasible to conduct a randomized controlled trial (RCT), due to practical and/or ethical reasons, making causal inference challenging. Rapid developments in the field of (substance use) genetics have led to the emergence of a powerful causal method that may solve these problems. Genetic variants robustly associated with a potential risk factor of interest, identified through GWAS, can be used as instrumental variables in an approach called ‘Mendelian randomization’ (MR) [81]. Substance use is an obvious modifiable risk factor that may be impacted by policy and preventive efforts, making MR for substance use disorders a particularly fruitful avenue to pursue [82].

MR relies on three core assumptions; the included genetic variants, the ‘genetic instrument’, should (1) be robustly associated with the exposure variable, (2) not be associated with confounders of the relationship between exposure and outcome, and (3) not be associated with the outcome through any other path than the exposure [83]. Depending on the relation of interest, there are additional assumptions [84]. To estimate the causal effect, Inverse-variance weighted (IVW) regression is conducted, which assumes no violation of the core assumptions. An important part of an MR study is to conduct sensitivity approaches to assess the robustness of a potential causal finding. For an extensive discussion about the theory and practical application of MR, we refer to two excellent reviews by Richmond and Davey Smith [84] and Davies et al. [83]. Examples of commonly applied sensitivity methods are: weighted median regression [85], weighted mode regression [86], MR-Egger [87], MR-PRESSO [88], and GSMR [89]. Apart from this selection of methods a wide range of other approaches exists (such as Bayesian MR [90] and an integration of MR and the direction of causation twin model [91]). Finally, a particularly useful addition to the MR ‘toolbox’ worth noting here is multivariable MR, where (an) additional exposure variable(s) can be added [92]. An important weakness of MR as it is often applied, is that it can be biased by assortative mating, dynastic effects, and population structure [93]. These biases can be overcome by using family-based GWAS estimates and conducting standard MR methods [93, 94] or by specific within-family MR methods [95].

**Fig. 4:**
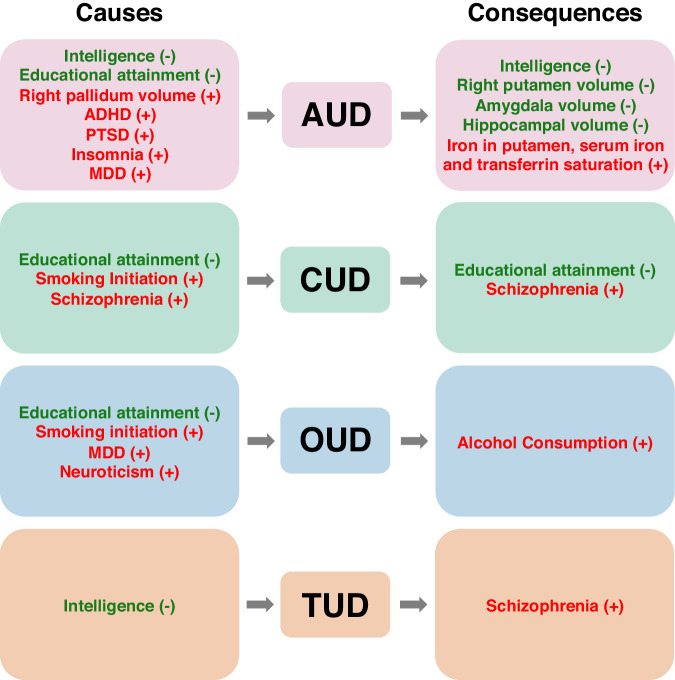
Putative causes and consequences of substance use disorders from Mendelian Randomization studies. This figure displays traits that have been found to causally influence the risk of major substance use disorders (“Causes”) and traits that are influenced by these disorders (“Consequences”). Negative directions of effect are displayed in green (−) while positive directions of effects are influenced in red (+). Note that this figure includes traits with a reported causal association with substance use disorders but does not consider the strength of evidence of causation or effect size of the association.

Mendelian randomization has been used to assess causal effects between SUDs and mental health, behavioural, and physical traits (Fig. 4). The most well-studied traits include cognitive functioning and educational attainment, structural brain measures, and psychiatric disorders (predominantly MDD, PTSD, ADHD). For alcohol use disorder, there is consistent evidence that higher intelligence and educational attainment causally decrease the risk of developing AUD [9, 96, 97]. One study indicated that PAU liability, which combined AUD and problematic drinking, may also causally decrease educational attainment [9], however other studies have failed to replicate this finding [97]. There is no evidence for causal effects in either direction between AUD and executive functioning [98], nor is there evidence for causal effects of alcohol dependence on late-onset Alzheimer’s disease risk [99]. While there was some evidence that AUD liability causes a later age of onset of Alzheimer’s, this is likely due to survival bias [99]. When focusing on more proximal measures, brain structure and other imaging phenotypes, there is evidence that a larger right pallidum volume increases AUD risk and in the other direction, that AUD liability decreases right putamen volume [100], amygdala volume, and hippocampal volume [101]. There is also some evidence that AUD increases markers of iron in the putamen [102] and basal ganglia [103], but this evidence is weak. Finally, with regards to psychiatric traits, there is no clear evidence of causal effects between AUD and loneliness [104], self-harm [105], or suicide [106]. While there is weak evidence of a causal effect of ADHD on AUD, but not the reverse, from one study [82] this was not replicated in another [66]. There is evidence for a causal effect of PTSD [107], insomnia [108], and of MDD [109] on AUD, but not the reverse.

For CUD there is evidence for bidirectional causal effects with educational attainment [110] but no evidence for causal effects with suicide [106]. In addition, a causal effect of CUD on schizophrenia has been identified [111], although we cannot rule out the presence of bidirectional effects [52, 112]. Finally, there is evidence that smoking initiation causally increases the risk of CUD, acting as a gateway [113].

Higher intelligence may decrease the odds of nicotine dependence [96] and nicotine dependence increases the risk of schizophrenia (even when corrected for CUD) [111]. There is no clear evidence for causal effects between nicotine dependence and ADHD [114], nor from nicotine dependence to suicide [106].

Finally, for opioid dependence there is no clear evidence for causality with suicide [106], weak evidence of an effect of opioid dependence on increased alcohol drinking [113], and evidence that smoking initiation, MDD, and neuroticism increase and higher educational attainment decrease opioid dependence risk [40]. For cocaine dependence there is no clear evidence for causal effects with other substance use behaviours [113] or ADHD [66].

Overall, MR studies have led to the identification of important, putative causal relations between substance use disorders and (poor) mental health outcomes. This knowledge should be considered in the development of preventive efforts and health messages and followed up on with more extensive analyses to pinpoint the exact causal pathways and mechanisms. An important approach to further improve the reliability of causal findings is ‘triangulation’ [115], that is, explicitly combining MR with other types of research method in a single study [116]. If the findings of different research methods, with different biases, all point to a causal effect, it is much less likely to be spurious. For instance, MR can be combined with observational (cohort) analyses, other genetically informative designs such as discordant twin or sibling analyses, or even natural experiments such as policy changes. Given its convenience and the ongoing expansion of even more sophisticated sensitivity methods, it is expected that MR will remain an important method in the field of causal inference.

### Pharmacogenomics of substance use disorders

There are several approved and effective pharmacological treatments of substance use disorders [117]. Incorporating genetic information into the treatment decision-making process may potentially improve patient management and outcomes [118]. Here, we will summarize the results of pharmacogenomics studies investigating how genes affect a person’s responses to pharmacological treatment, with a specific focus on alcohol, tobacco and opioid use disorders.

Several medications have demonstrated efficacy for the treatment of AUD, but pharmacological agents remain underutilized [119] and are only moderately effective with large variation in effects across patients. Pharmacotherapy for AUD targets neurotransmitter systems that are impacted by the consumption of alcohol, including opioidergic, dopaminergic, GABAergic, glutamatergic, and serotonergic neurotransmission [119]. Three medications are approved by the U.S. Food and Drug Administration (FDA) for the treatment of AUD: disulfiram, acamprosate, and naltrexone. There are also several drugs that are used off-label to treat AUD, including topiramate, gabapentin, baclofen, ondansetron and varenicline, among others [120].

Two recent reviews have summarized findings from pharmacogenetic studies for approved and off-label medications [119, 121]. The overall conclusion is that pharmacogenetic results are insufficient to inform clinical practice, due to small sample sizes and a lack of standardized trial designs and outcome measures. The most widely studied genetic polymorphism is rs1799971 as a potential mediator of naltrexone; this SNP encodes a non-synonymous substitution (Asn40Asp) in the mu-opioid receptor gene, OPRM1. A meta-analysis including seven RCTs found that the Asn40Asp SNP has a nominally significant moderating effect on drinks per day, while no significant associations were found for four other outcomes [122]. Therefore, the evidence for the utility of this SNP as a predictor of naltrexone response remains inconclusive. A summary of pharmacogenetic findings for the remaining medications is provided in Table 1.

**Table 1:** Summary of clinical and pharmacogenetic studies of substance use disorders.

Disorder	Drug	Pharmacogenetics summary
Alcohol use disorder^a^	Disulfiram	Results are limited by small sample sizes
	Naltrexone	rs1799971 has been suggested as a potential mediator of naltrexone; this SNP encodes a non-synonymous substitution (Asn40Asp) in the mu-opioid receptor gene, OPRM1. A meta-analysis including seven Randomized Controlled Trials (RCTs) showed that the Asn40Asp SNP shows a nominally significant moderating effect on drinks per day, while no significant associations were found for four other outcomes [122].
	Injectable naltrexone	None
	Acamprosate	Acamprosate treatment efficacy may be partially moderated by genetic variation of genes regulating stress and reward pathways, including GATA4, DRD2, GABRA6, GABRB2 and GRIN2B
	Topiramate	Results are limited by small sample sizes
	Gabapentin	None
	Baclofen	Pharmacogenetic data are limited to date and focused on GABA receptors
Tobacco use disorder	Nicotine-replacement therapies	A meta-analysis of 18 trials (*N* = 9017) showed that non-Hispanic black smokers with rs16969968-GG genotypes were 3.5-fold and 5.8-fold more likely to abstain at 6 months and at end of treatment, respectively [128].
	Bupropion	The CYP2B6*6 allele and genotype-determined CYP2B6 poor and intermediate metabolizer phenotypes were found to be associated with significantly lower total active moiety and reduced exposures to HB [130]. A meta-analysis of trials of smoking cessation pharmacotherapies did not reveal any significant associations [128].
	Varenicline	Results are limited by small sample sizes
Opioid use disorder	Methadone maintenance therapy (MMT)	The most reproducible result is an association between the CYP2B6*6 allele and (S)-methadone plasma concentrations [134]. However, a meta-analysis of 7 articles did not find a significant association between CYP2B6*6 and methadone response [133].

^a^Results are partly adapted from [121].

While the majority of the pharmacogenetic studies have focused on candidate genes, a genome-wide pharmacogenomics study of acamprosate and naltrexone included 1083 European ancestry participants [74]. This study identified genetic variants near the PTPRD gene influencing time until drinking relapse in naltrexone-treated patients [74].

A combination of behavioural support and pharmacotherapy maximizes chances of successful long-term cessation of tobacco use. The most common pharmacological treatments of Tobacco Use Disorder are nicotine-replacement therapies, bupropion (a non-tricyclic antidepressant), and varenicline (a selective nicotinic receptor partial agonist). Meta-analyses have confirmed the efficacy of these therapies on abstinence at 6-month or longer follow-up, with varenicline being the superior treatment [123]. A large proportion of the ability to quit smoking is heritable (50–60%) [124]. Smokers with genetically slow nicotine metabolism have higher cessation success on behavioural counselling and nicotine patches compared with smokers with genetically fast nicotine metabolism [125].

Nicotine is primarily metabolized by CYP2A6, and variability in rate of metabolism contributes to vulnerability to tobacco dependence, and response to smoking cessation treatment [126]. El-Boraie and Tyndale have reviewed the results of pharmacogenomics studies on various smoking phenotypes [127]; here, we will focus primarily on summarizing data for genetic variants associated with smoking cessation and a genetically informed biomarker of nicotine clearance, the Nicotine Metabolite ratio (NMR).

### Nicotine-replacement therapy

A meta-analysis of 18 trials (*N* = 9017) was conducted in 2019, including 40 active (bupropion, nicotine-replacement therapy [NRT], varenicline, or combination therapies) versus placebo comparisons and 16 active versus active comparisons [128]. Data were available for nine SNPs in five genes, two variable number of tandem repeat polymorphisms, and the NMR biomarker. The study revealed that non-Hispanic black smokers with rs16969968-GG genotypes were 3.5-fold and 5.8-fold more likely to abstain at 6 months and at end of treatment, respectively. This nonsynonymous variant is within the alpha-5 nicotinic acetylcholine receptor (*CHRNA5*) gene, and is strongly associated with smoking quantity and nicontine dependence in large-scale GWAS and functional studies [27, 49]. No evidence of rs16969968 effect modification was observed for non-Hispanic white smokers except for short-term outcomes. There was no clear statistical evidence for other genotype-by-treatment combinations nor for an association between the NMR and treatment outcomes. Most pharmacogenomics studies have focused on the role of one or more candidate genes, while treatment response is likely under polygenic influences of multiple variants with small and large effects. An elegant way to estimate the combined effect of multiple genetic variants is PGS analysis - Uhl et al. developed a PGS based on 12,058 SNPs and showed that this score significantly predicted ability to quit after nicotine patch treatment, with 43 vs 13% quit in the upper vs lower PGS terciles [129].

### Bupropion

This dopamine and norepinephrine reuptake inhibitor and nicotine antagonist is widely used to treat depression. It is also one of the first-line pharmacotherapy options for smoking cessation [130]. Bupropion is metabolized to its active metabolite, hydroxybupropion (HB), by the genetically polymorphic cytochrome P450 2B6 (CYP2B6) enzyme [130]. Eum et al. meta-analyzed the results of ten studies (*N* = 413) evaluating the influence of *CYP2B6* polymorphisms on bupropion exposure and on hydroxybupropion (HB), one of the three main active metabolites of bupropion and an important component for pharmacological activity and therapeutic effectiveness of the compound. The authors showed that the CYP2B6*6 allele and genotype-determined CYP2B6 poor and intermediate metabolizer phenotypes are associated with significantly lower total active moiety and reduced exposures to HB [130]. However, a meta-analysis of trials of smoking cessation pharmacotherapies did not reveal any differences between genotypes and efficacy of bupropion treatment outcomes, including 6-month abstinence, or end-of treatment abstinence [128]. However, the authors argued that the lack of significant associations may have been due to sample size limitations [128].

### Varenicline

This partial agonist of nicotinic receptors in the central nervous system acts to relieve cravings and withdrawal symptoms as well as reducing the rewarding effect of smoking [123]. Varenicline undergoes almost no metabolism, but variation in the transport of varenicline throughout the body may alter treatment efficacy [125]. Similar as for bupropion, a meta-analysis of clinical trials did not reveal any differences between genotypes and efficacy of varenicline treatment outcomes, including 6-month abstinence, or end-of treatment abstinence [128]. There is some evidence that varenicline is more effective in those with rs16969968 GA/AA genotypes compared to GG genotypes in African American smokers although this finding will need to be replicated [131]. Another study found an association between CYP2B6 rs8109525 and varenicline efficacy [132], but this association did not survive correction for multiple testing and further replication is required.

Methadone maintenance therapy (MMT) is a substitute opioid therapy used to treat opioid withdrawal symptoms and is considered to be the most effective treatment for opioid addiction [133]. Genetic variability contributes to the effectiveness of MMT. Another commonly used pharmacotherapy for the treatment of OUD is buprenorphine, a weak MOR agonist and a partial kappa-opioid receptor antagonist, although buprenorphine seems to be less effective than MMT due to reduced retention in treatment [134]. A GWAS of buprenorphine treatment response revealed 6 nominally significant loci, four of which were located near previously characterized genes: rs756770 (ADAMTSL2), rs11782370 (SLC25A37), rs7205113 (CRISPLD2), and rs13169373 (LINC01947) [135]. Crist et al. reviewed pharmacogenetic findings of OUD treatment [134]. We refer the reader to this review for a full review of pharmacogenetic findings of OUD treatment dose and treatment response. In summary, due to a lack of large data sets with outcome and genotype data, there are no robust and replicable associations between genetic variants and OUD treatment outcomes. The most reproducible result is an association between the CYP2B6*6 allele and (S)-methadone plasma concentrations [134]. However, a meta-analysis of 7 articles did not find a significant association between CYP2B6*6 and methadone response [133].

### Clinical implications and future work

Large-scale genetic studies have revolutionized the discovery of genetic loci associated with substance use disorders, especially for AUD, OUD, and TUD, while relatively few loci have been identified for CUD due to smaller sample sizes. The early promises of GWAS included the development of new and effective treatments and improved patient stratification. The availability of well-powered GWAS is a first step towards achieving these aims, although several barriers need to be overcome [45]. PGS explain around 2–6% of the phenotypic variation for AUD, OUD, and TUD and PGS can be used for risk stratification, diagnosis and treatment of substance use disorders, when used in combination with established risk measures [136]. In addition, the integration of GWAS data with molecular phenotypes may inform the identification of new and biologically relevant drug targets for substance use disorders through computational drug repurposing [45]. Future studies can be strengthened by the development of drug–gene databases across diverse cell types, including neuronal and other types of brain cells. In addition, new approaches have been developed to model the phenotypic complexity both within and across substance use disorders and will need to be applied at the widest possible scale, for example, by extending analyses to biobanks, such as the impending Global Biobank Meta-analysis Initiative [137].

## Supplementary information

Supplementary Tables
